# Emotional regulation neural circuitry abnormalities in adult bipolar disorder: dissociating effects of long-term depression history from relationships with present symptoms

**DOI:** 10.1038/s41398-020-01048-1

**Published:** 2020-11-02

**Authors:** Michele A. Bertocci, Jeffrey Bergman, Joao Paulo Lima Santos, Satish Iyengar, Lisa Bonar, Mary Kay Gill, Halimah Abdul-waalee, Genna Bebko, Richelle Stiffler, Jeanette Lockovich, Haris Aslam, Cecile Ladouceur, John Merranko, Rasim Diler, Boris Birmaher, Amelia Versace, Mary L. Phillips

**Affiliations:** 1 Department of Psychiatry, Western Psychiatric Hospital, University of Pittsburgh School of Medicine, University of Pittsburgh, Pittsburgh, PA USA; 2 Department of Statistics, University of Pittsburgh, Pittsburgh, PA USA

**Keywords:** Bipolar disorder, Prognostic markers

## Abstract

Bipolar disorder (BD) is common and debilitating and confounding effects of depression history on neural activity in BD are unknown. We aimed to dissociate neural activity reflecting past depression-load vs. present symptom severity using the Course and Outcome of Bipolar Youth (COBY), a prospective longitudinal cohort study of pediatric-onset BD. In *n* = 54 COBY (18–32 years), we modeled depression scores over time (up to 17.5 years) using a standardized autoregressive moving average (ARMA) model, followed by *k*-means cluster analysis. *N* = 36 healthy participants (HC, 20–36 years) were included. Using two factorial analyses, we parsed the impact of ARMA-defined past depression-load on neural activity from the impact of current symptoms on neural activity (*p* < 0.001, *k* > 30) and examined relationships with past and present symptoms (*p*s FDR-corrected). ARMA identified three COBY groups based on past depression-load. ARMA-defined COBY participants with the greatest past depression-load vs. other groups showed greater activity in right temporoparietal junction, thalamus, insula, premotor cortex, left fusiform gyrus, bilateral precuneus and cerebellum. In contrast, BD-COBY participants vs. HC showed greater activity in left hippocampus, dorsolateral prefrontal cortex, and right somatosensory cortex, plus the above thalamus, premotor cortex and cerebellum; activity positively correlated with present symptom severity in most regions. Past depression-load was related to social cognition and salience perception network activity, potentially reflecting heightened attention to socially relevant distracters, while present symptoms were associated with emotion processing and reappraisal network activity, potentially reflecting abnormal emotional experience and regulation. Differentiating aberrant neural activity related to long-term depression vs. present affective symptoms can help target interventions to networks associated with pathophysiological processes, rather than long-term illness effects.

## Introduction

Bipolar disorder (BD) is a complex psychiatric illness characterized by affective lability and emotional dysregulation, associated with depression and mania. Over time, depressive episodes in BD often become more frequent, and of longer duration^1^, probably because prescribed medications are often more effective for hypo/manic than depressive episodes^2,3^. Indeed, depressive episodes in BD are usually 50% longer than manic episodes^4^. This increase in the magnitude and severity of depression over time is associated with premature death, functional impairment, medical and psychiatric comorbidities, reduced quality of life^5^ and an estimated annual cost of $202 billion^6^.

A growing number of studies has aimed to identify neural circuity abnormalities in BD in order to elucidate underlying pathophysiological processes to facilitate identification of neural targets to guide the development of new interventions for BD (e.g., neuromodulation, and novel cognitive behavior therapies (CBT) and medications). Findings from these studies indicate patterns of significantly greater and lower activity in BD vs. healthy adults in several neural regions implicated in emotion regulation, including prefrontal cortex^7–12^, amygdala^7,9,10^, insula, thalamus, and hippocampus^11,12^ in individuals with BD. Yet, the potentially confounding effect of long-term depression, and other factors, such as long-term medication, on neural circuitry supporting key processes relevant to understanding the pathophysiology of BD, including emotional regulation, are not clearly understood and have not, to our knowledge, been examined. Dissociating past depression-load from present symptom-related patterns of abnormal emotional regulation neural activity in BD is thus an important goal in neuroimaging research in BD. Meeting this goal will first provide a broader understanding of the pathophysiological processes related to different past depression trajectories in BD. Meeting this goal will also facilitate appropriate targeting of neuromodulation interventions to neural regions implicated specifically in pathophysiological processes of BD, vs. targeting these interventions to neural regions in which abnormal activity reflects long-term illness effects. To date, these goals have been difficult to achieve because of limited information regarding long-term past psychiatric history in most neuroimaging studies of BD.

The Course and Outcome of Bipolar Youth (COBY) study is a prospective naturalistic examination of pediatric-onset (<18 years) BD, in which a large cohort has been continually followed since 2001 with semiannual assessments^13^. COBY provides the opportunity, for the first time to our knowledge, to recruit adults with a well-documented and long history of childhood-onset BD into neuroimaging studies in order to dissociate past from present pathology.

We recruited participants from COBY in order to test three aims: (1) to summarize using autoregressive moving average (ARMA) individual-level long-term past depression-load in adults with pediatric-onset BD; (2) to identify patterns of abnormal emotional regulation-related whole-brain activity associated with past depression-load; and (3) to identify patterns of emotional regulation-related neural activity associated with present BD symptom (depression, mania and anxiety) severity in COBY participants. After using ARMA models to calculate individualized past depression-trajectories, and *k*-means clustering to classify COBY participants into groups characterized by the degree of long-term past depression-load, we hypothesized that relative to BD-COBY participants with lower past depression-load, BD-COBY participants with greater past depression-load would show a greater magnitude of abnormal activity in neural circuitries relevant to BD, including emotional regulation circuitry^10^; and patterns of past depression-load-related abnormal neural activity would be distinct from patterns of abnormal neural activity associated with present BD symptom severity in COBY participants.

## Methods

### Participants

Pediatric-onset BD COBY participants (*n* = 54) were clinically followed for up to 17.5 years prior to neuroimaging assessment; weeks prior to scan: range = 104–908 weeks mean (standard deviation (SD)) = 696.9 (164.5), age: range = 18.9–32.7, mean (SD) = 25.60 (4.0), 42 females. Thirty-six statistically matched Healthy adult participants (HC) were used as the comparison group (12 newly recruited participants and 24 from the Dimensions of Affect, Mood, and Neural Activity Associated with Distress study, R01MH100041) mean age = 25.92 (4.96), 19 females (Table 1). Institutional Review Boards approved both studies and all participants gave the consent to participate.

**Table 1:** Clinical and demographic information.

	BD	HC		
	*n* = 54	*n* = 36	Test statistic	*p*
Age	25.60 (4)	25.9 (5)	*t*(87) = 0.334	0.839
Sex (female)	27	19	chi2 = 0.029	0.865
IQ	102.4 (11.1)	104.3 (13.3)	*t*(39) = −0.49	0.626
Diagnosis at scan				
Bipolar disorder I	49	0	n/a	
Bipolar disorder II	5			
Anxiety disorder	24	0	n/a	
ADHD	17	0	n/a	
Substance use disorder	16	0	n/a	
Years in study	13.4 (3.16)			
Present-scan-day medication	11	0	n/a	
Antidepressant/ mood stabilizer	7	0	n/a	
Antipsychotic	6	0	n/a	
Stimulant	3	0	n/a	
High/persistent past medication use				
Antidepressant	20	0	n/a	
Lithium	15	0	n/a	
Non-lithium mood stabilizer	13	0	n/a	
Antipsychotic	18	0	n/a	
Stimulant	15	0	n/a	
Study intake information		0	n/a	
Age of bipolar onset	8.41 (3.7)	0	n/a	
Duration of illness at intake	4.02 (2.5)	0	n/a	
Lifetime diagnosis				
Generalized anxiety disorder	14/51	0	n/a	
ADHD	33/51	0	n/a	
Conduct disorder	4/51	0	n/a	
Oppositional defiant disorder	25/51	0	n/a	
Substance Use disorder	3/51	0	n/a	
CGAS range 41–85	60.02 (10.5)	0	n/a	

See Supplementary information for comparisons between clusters of significant activity with BD subtype: BD type I (BDI) and BD type II (BDII) (Supplementary Tables 1 and 2); and present-scan-day and historical medication (Supplementary results). Data are mean (standard deviation) or count as appropriate.

*CGAS* Children’s Global Assessment Scale, *ADHD* attention deficit hyperactivity disorder.

### Clinical assessments

At each follow-up, COBY participants were assessed for longitudinal changes in DSM-IV symptoms and functioning using the A‐LIFE Psychiatric Rating Scale (PSR)^14^. For this analysis we focused on depression, mania, hypomania, and generalized anxiety symptoms with PSR scores ranging from 1 to 6; 1–2 = no/minimal symptoms, to 3–4 = varying levels of subthreshold symptoms and impairment, and 5–6 = full DSM‐IV criteria, with 6 as the most severe and impaired. Medication use was recorded at each assessment. As a longitudinal measure of mania and depression severity over the follow-up period, we assessed the most severe episode between follow-up interviews using the schedule for affective disorders and schizophrenia for school-age children (KSADS) mania and depression rating scales^15^, respectively.

(See Supplementary information for description of A-LIFE PSR assessment over time, exclusion criteria, neuroimaging data acquisition, the emotional distracter-n-back (implicit emotional regulation) neuroimaging task, and data processing.) Briefly, the main conditions of interest for the emotional regulation task consisted of high memory load (2-back—e.g., press the button whenever the presented letter is identical to the letter present two trials back (L-X-L)) each flanked with an emotional face distracter condition (fearful, happy, or neutral face distracter).

### Present-scan-day symptoms

To measure present-scan-day depression, manic, and anxiety symptom severity, all participants completed on scan-day, respectively, the Hamilton Depression Rating Scale ^16^, Young Mania Rating Scale ^17^, and the Spielberger State Trait Anxiety Inventory (Adult Version)^18^.

### Data analysis

#### Aim 1

Weekly depressive symptoms were collected for up to 17.5 years prior to fMRI scanning, using the PSR scale (range: 1–6) in the A-LIFE. Historically, data collection was scheduled semiannually until the most recent funding cycle, which scheduled data collection every 18 months (mean time between assessments = 12.5 months (range: 2.07–41.95 months)). Given that data were timeseries with serial dependence, and the large volume of individual-level data (up to 908 datapoints), we utilized autoregressive moving average (ARMA) to summarize individual weekly depression scores, we utilized the augmented Dickey−Fuller test for stationarity. ARMA models are a validated approach for analyzing stationary timeseries data (consistent mean and variance over time) (see Supplementary information). We used the “arima() in R stats package” to generate the ARMA models^19^. Given the individual nature of ARMA models and the interest in group-level information, we used *k-means clustering* to group the autoregressive (AR) coefficients that reflects the “memory” of the model and can inform our understanding of depression history. Clustering/grouping of the AR coefficients was performed by comparing the results of 2, 3, 4, and 5 *k-means* clusters. To validate this approach, we then compared the number of DSM-IV-criteria-defined depressive episodes for individuals in each ARMA-defined-COBY group.

Mania, hypomania, and generalized anxiety history loads were explored using percent of recorded time with a threshold PSR score. Past manic load (PSR score ≥ 5; range = 0−0.14 of total weeks; mean (SD) = 0.01 (0.023) of total weeks), past hypomanic load (PSR score ≥ 5; range = 0−0.935 of total weeks; mean (SD) = 0.04 (0.14) of total weeks), and past anxiety load (PSR score ≥ 5; range = 0–1 of total weeks; mean (SD) = 0.16 (0.25) of total weeks).

Past depression and mania severity trajectories were calculated using Mplus^20^ (number of follow-up assessments range: 5–24 assessments).

Variables not meeting model assumptions were transformed, or nonparametric tests were used.

#### Aim 2

To identify wholebrain activity related to depression-load groups, a full factorial model (number of ARMA-defined COBY groups and HC by 3, 2-back emotional face distracter conditions: 2-back fear, 2-back happy, 2-back neutral) was performed in SPM12 (F > 8.0, *p* < 0.001, *k* > 30). Parameter estimates were extracted from clusters showing a significant main effect of group, and/or group-by-condition interaction, in the full factorial model. ANCOVA, covaried for age and gender, and pairwise comparisons evaluated the direction of between-group differences in activity in these clusters to each condition. FDR-corrected threshold^21^ accounted for the number of parallel between-group tests.

#### Aim 3

To identify significant differences in wholebrain activity between BD-COBY participants (as a whole) and HC, we compared the BD-COBY sample (*n* = 54) and HC (*n* = 36) (2 groups by 3, 2-back emotional face distracter conditions: 2-back fear, 2-back happy, 2-back neutral), using SPM12 (*p* < 0.001, *k* > 30). Parameter estimates were extracted from clusters showing a significant main effect of group, and/or group-by-condition interaction, in the full factorial model. ANCOVA, covarying for age and gender, evaluated the direction of between-group/interaction effects. We then examined, across all participants, relationships between present-scan day depression, mania, and anxiety severity and extracted parameter estimates from the clusters of activity that differed between groups or showed an interaction, using correlation analyses, and FDR-corrected threshold^21^ for the number of parallel cluster-symptom relationships, accounting for the number of BD-COBY vs. HC between-group tests in these clusters.

### Specificity analyses

Visual inspection of the overlap and differences in neural maps generated from each group-level analysis was performed using xjview (https://www.alivelearn.net/xjview).

To assess the specificity of our findings, correlation or *t* test analyses examined: (1) relationships between main effect of group or interaction activity in the ARMA-defined-COBY group vs. HC model and present-scan-day depression, mania and anxiety severity, with FDR-corrected threshold^21^; and (2) main effect of group or interaction activity in the BD-COBY participant vs. HC model and membership derived from the ARMA-defined-COBY analysis, with FDR-corrected threshold^21^.

#### Exploratory analyses

*t* tests and correlational analyses examined the effects of medication (present-scan-day/past medication use), mood disorder age-of-onset, lifetime comorbid history (i.e., generalized anxiety disorder (GAD), attention deficit hyperactivity disorder (ADHD), conduct disorder (CD), oppositional defiant disorder (ODD)), illness history loads (mania, hypomania, and generalized anxiety), and depression and hypo/mania severity trajectories on extracted parameter estimates from activity differing between groups in both of the above analyses.

## Results

### Aim 1

We standardized the ARMA parameters for each participant (AR = 4, MA = 2). The four AR “memory” coefficients optimally produced three groups from *k-means clustering*; all clusters *p* < 0.001. We additionally compared the ARMA-defined groups with a classical definition of depression severity (numbers of past clinically depressive episodes) to confirm the use of our approach and to aid the understanding of findings. ARMA-defined COBY Group 1 showed the lowest past depression-load (*n* = 27): a range of 0–7 depressive episodes (mean = 1.70 (1.81) episodes). ARMA-defined COBY Group 2 (*n* = 12) had a range of 0–8 episodes (mean = 2.25 (2.26) episodes). ARMA-defined COBY Group 3 showed the greatest past depression-load (*n* = 15): range of 1–11 episodes (mean = 4.13 (3.09) episodes). ARMA-defined COBY Group 3 reported significantly more past depressive episodes than ARMA-defined COBY Group 1 and Group 2 (F(2,51) = 5.36, *p* = 0.008). ARMA-defined COBY Groups 1 and 2 did not differ significantly in the number of past depressive episodes.

### Aim 2

There was a main effect of group (three ARMA-defined COBY groups and HC) on activity during all 2-back with face distracter conditions (2-back fear, 2-back happy, 2-back neutral): right temporoparietal junction (F(3,258) = 16.24); right thalamus (F(3,258) = 10.35); right premotor cortex (F(3,258) = 8.94); left fusiform gyrus (F(3,258) = 9.67); right insula (F(3,258) = 4.03); and bilateral precuneus (BA7; left:F(3,258) = 8.59, right: F(3,258) = 12.40), cerebellum (left: F(3,258) = 9.24, right: F(3,258) = 10.48 and right: F(3,258) = 10.45); all Fs > 8.0, *p* values < 0.001, *k* > 30. There was no significant activity for the group-by-condition interaction.

**Fig. 1:**
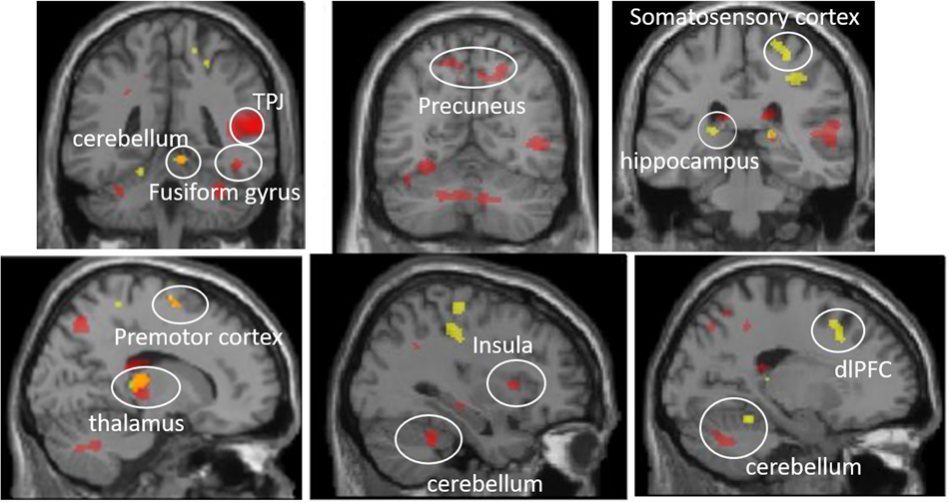
Neural regions from aims 2 and 3. Red represents ARMA-based groups and Healthy comparisons; unique regions were right temporoparietal junction (TPJ), right insula, left fusiform gyrus, bilateral precuneus, and subregions of the cerebellum. Yellow represents COBY and Healthy participants comparisons; unique regions were left hippocampus, dorsolateral prefrontal cortex (dlPFC), and right somatosensory cortex. Orange represents the overlapping regions between the two analyses; overlapping regions were right thalamus, premotor cortex, and cerebellum.

ANCOVA and *t* tests on extracted parameter estimates covarying for age and gender (FDR-corrected *p* value = 0.010684^21^; 10 clusters-by-6 between-ARMA-defined-group tests = 60 tests) revealed significantly greater activity predominantly for ARMA-defined Group 3 vs. other groups in the majority of clusters (Fig. 1 and Table 2).

**Table 2:** ANCOVA comparisons with pairwise tests of extracted parameter estimates from significant clusters comparing ARMA-defined groups and Healthy participants controlling for age and gender.

						ANCOVA results							
	SPM coordinates and cluster size					ARMA-defined and healthy group		Age		Gender			
Neural region		*x*	*y*	*z*	*k*	F(3,84)=	*p*=	F(1,84)=	*p*=	F(1,84)=	*p*=	Post hoc test	
Thalamus	R	14	−26	2	217	**8.01**	<**0.001**	0.13	0.724	0.32	0.574	G3 > HC	G1 > HC
Temporoparietal Junction	R BA 22	56	−40	12	1574	**5.40**	**0.002**	2.13	0.149	2.16	0.146	G3 > G1, HC	
Cerebellum	L	−34	−42	−32	31	3.60	<0.017	1.76	0.188	3.05	0.084		
Cerebellum	R	36	−44	−30	83	**5.21**	**0.002**	0.01	0.909	0.19	0.663	G3 > HC	
Cerebellum	R	8	−58	−34	428	2.98	0.036	0.38	0.539	0.16	0.693		
Insula	R	34	14	6	45	**6.73**	<**0.001**	1.49	0.226	0.76	0.386	G3 > G1, HC	G2 > G1, HC
Fusiform gyrus	L	−28	−64	−12	344	**9.90**	<**0.001**	0.37	0.544	0.03	0.874	G3 > G1, HC	
Premotor cortex	R BA 6	18	2	64	117	**6.93**	<**0.001**	1.12	0.293	2.33	0.131	G3 > G1, HC	G2 > HC
Precuneus	L BA 7	−8	−50	66	89	**8.13**	<**0.001**	1.01	0.319	3.05	0.084	G3 > G1, HC	G1 > HC
Precuneus	R BA 7	20	−68	52	242	3.58	0.017	0.78	0.380	0.00	0.968		

Coordinates in MNI space; *k* = number of voxels in region; Bold indicates significant test. FDR significance threshold < 0.010684.

*G3* ARMA-defined Group 3, *G2* ARMA-defined Group 2, *G1* ARMA-defined Group 1, *HC* Healthy participants, *R* right, *L* left, *BA* Brodmann area.

### Aim 3

BD-COBY relative to HC had greater activity in left dorsolateral prefrontal cortex (dlPFC F(1,264 = 13.05), left hippocampus (F(1,264) = 15.86), right thalamus (F(1,264) = 22.29), right somatosensory cortex (F(1,264) = 14.76) and right premotor cortex (F(1,264) = 17.82), and bilateral cerebellum (left: F(1,264) = 16.91; right: F(1,264) = 15.83) during all 2-back with face distracter conditions (2-back fear, 2-back happy, 2-back neutral): all *p*s < 0.001, *k* > 30. There was no significant activity for the group-by-condition interaction.

**Fig. 2:**
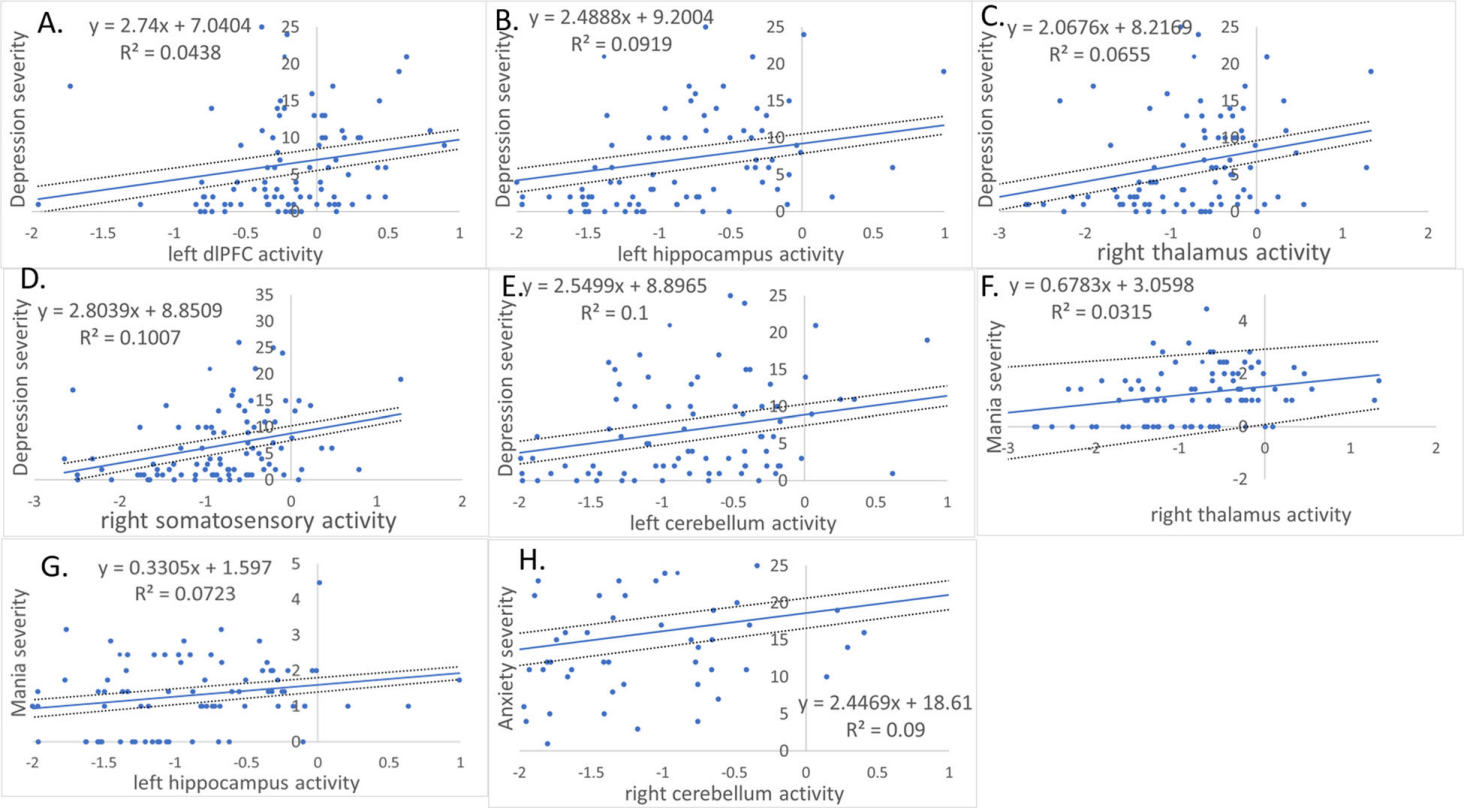
Linear relationships between present affective symptom severity and neural activity distinguishing COBY participants from HC. **A** Relationship between left dlPFC activity and depression severity; **B** Relationship between left hippocampus activity and depression severity; **C** relationship between right thalamus activity and depression severity; **D** relationship between right sensorimotor cortex activity and depression severity; **E** relationship between left cerebellum activity and depression severity; **F** relationship between right thalamus activity and mania severity and **G** relationship between left hippocampus activity and mania severity; **H** relationship between right cerebellum and anxiety severity. Blue lines represent the trend lines and the dotted lines represent the 95% confidence intervals. These relationships were for Spearman’s correlations for depression and mania due to nonparametric distributions of these affective symptom scores.

ANCOVA and *t* tests on extracted parameter estimates confirmed the above direction of the between-group findings, covarying for age and gender. In all BD-COBY participants, present-scan-day depression severity was positively associated with left hippocampal (rho = 0.41, *p* ≤ 0.001), right thalamus (rho = 0.29, *p* = 0.006), left cerebellum (rho = 0.36, *p* ≤ 0.001), left dlPFC (rho = 0.28, *p* = 0.007), and right somatosensory cortical (rho = 0.33, *p* = 0.002) activity. Right thalamus (rho = 0.27, *p* = 0.008) and left hippocampus (rho = 0.27, *p* = 0.010) activity were positively associated with present-scan-day mania severity (Fig. 2). Right cerebellum activity (*r* = 0.300, *p* = 0.004) was positively associated with present-scan-day anxiety severity. An FDR corrected *p* value = 0.01273^21^ was used (7 clusters-by-3 symptom relationship + 7 BD-COBY vs. HC between-group tests = 28 tests; Fig. 1 and Table 3).

**Table 3:** ANCOVA comparisons with pairwise test of extracted parameter estimates controlling for age and gender comparing COBY (*n* = 54) vs. Healthy participants (HC) (*n* = 36).

						ANCOVA results						
	SPM coordinates and cluster size					Diagnostic group		Age		Gender		
Neural region		*x*	*y*	*z*	*k*	F(1,86)=	*p*=	F(1,86)=	*p*=	F(1,86)=	*p*=	Post hoc test
Somatosensory cortex	R BA 1	38	−28	44	453	**18.35**	**<0.001**	3.18	0.078	0.51	0.479	COBY > HC
Thalamus	R	12	−26	10	134	**16.72**	**<0.001**	0.01	0.944	0.20	0.654	COBY > HC
dlPFC	L BA 9	−24	30	38	130	**16.55**	**<0.001**	1.48	0.227	0.07	0.792	COBY > HC
Premotor cortex	R BA 6	18	2	64	68	**13.16**	**<0.001**	1.87	0.175	3.70	0.058	COBY > HC
Cerebellum	L	−18	−46	−18	38	**19.92**	**<0.001**	0.35	0.554	1.19	0.277	COBY > HC
Cerebellum	R	8	−44	−10	36	**15.11**	**<0.001**	5.36	0.023	**6.12**	**0.010**	COBY > HC
Hippocampus	L	−22	−34	10	40	**12.82**	**0.001**	1.60	0.209	0.10	0.759	COBY > HC

Coordinates in MNI space; *k* = number of voxels in region; bold indicates significant test. FDR significance threshold < 0.01273.

*dlPFC* dorsolateral prefrontal cortex, *R* right, *L* left, *BA* Brodmann area.

### Specificity analyses

Comparisons between the maps generated from the two analyses showed overlap in activity in several regions, including right thalamus, right premotor cortex, and right cerebellum. Activity associated uniquely with ARMA-defined-COBY-past depression-load was in right temporoparietal junction, right insula, left fusiform gyrus, bilateral precuneus and subregions of the cerebellum. Activity associated uniquely with BD-COBY diagnosis was in left hippocampus and dlPFC, and right somatosensory cortex (Fig. 1).

Specificity analyses of relationships among present-scan-day symptom severity and the ten clusters of activity showing a main effect of ARMA-defined COBY group in the 4-by-3 ANOVA in all participants (FDR-corrected *p* value = 0.009639 ^21^: 10 clusters-by-3 symptoms = 30 tests, 10 COBY vs. HC between-group tests, and 60 between-ARMA-defined group tests above = 100 tests) revealed significant associations among present-scan-day depression severity and right thalamus (rho = 0.33, *p* = 0.002), left precuneus (rho = 0.33, *p* = 0.002), and bilateral cerebellum (left: rho = 0.34, *p* = 0.001, right: rho = 0.29, *p* = 0.006) activity; and present-scan-day mania severity and right thalamus (rho = 0.32, *p* = 0.002) and right cerebellum (rho = 0.29, *p* = 0.006) activity. Anxiety severity was not associated with activity in these clusters (Supplementary Table 3).

Specificity analyses of between-group differences among ARMA-defined COBY group membership in the seven clusters of activity showing a main effect of BD-COBY participants vs. HC in the 2-by-3 ANOVA (FDR-corrected *p* value = 0.01035^21^: 7 clusters-by-6 ARMA-defined between-group comparisons = 42 tests, 7 clusters-by-3 symptoms = 21 symptom-cluster tests above, and 7 COBY vs. HC between-group tests = 70 tests) revealed no significant between-group membership differences (all *p*s > 0.046; Supplementary Table 4).

#### Exploratory analyses

Past and present-scan-day medication use were associated with either lower activity or showed no association in regions showing a main effect of group in both models (Supplementary information, Supplementary Tables 5 and 6, liberal *p* value threshold 0.05).

For ARMA-defined COBY vs. HC model main effect of group neural activity, there was a positive relationship between left fusiform activity and hypomania history load (*r* = 0.30, *p* < 0.028). There were no relationships with illness history loads of mania or GAD, illness severity trajectories, age of mood disorder onset, or lifetime comorbid diagnosis of ADHD, GAD, CD or ODD.

For main BD-COBY vs. HC model effect of group neural region, there was a positive relationship between right cerebellum activity and hypomania history load (*r* = 0.29, *p* = 0.035). COBY participants with a lifetime history of comorbid ADHD showed higher left cerebellum activity relative to those without lifetime comorbid diagnosis of ADHD (*t*(49) = −2.53, *p* = 0.015). There was a negative relationship between left dlPFC activity and age of onset of mood disorders (*r* = −0.30, *p* = 0.031) and COBY participants with lifetime comorbid CD diagnosis showed lower left dlPFC activity relative to those without lifetime comorbid CD (*t*(49) = 2.46, *p* = 0.018). There were no relationships with illness history loads of mania or GAD, illness severity trajectories, or lifetime comorbid diagnosis of GAD or ODD.

## Discussion

We aimed to dissociate patterns of abnormal neural activity associated with present-scan-day symptom severity from those patterns of abnormal neural activity that are effects of long-term depression in the context of BD. This is a critical step to ultimately enable appropriate targeting of interventions such as neuromodulation to neural regions that are implicated in pathophysiological processes associated with the development of BD, rather than targeting such interventions to neural regions in which abnormal activity represents effects of long-term illness. COBY provided a unique sample of BD adults in whom to separate effects of well-characterized depression history from present-scan-day symptom severity-related patterns of abnormal neural activity during emotional regulation. We identified patterns of abnormally elevated emotional regulation-related activity across all face distracter conditions in networks supporting visual social cognition and salience perception, and reappraisal and experience of emotion, that were associated, respectively, with past depression-load and present-scan-day affective symptom severity. Abnormally elevated activity in a cerebello-thalamic-premotor cortical network was associated with both past depression-load and present-scan-day symptoms.

To identify groups of COBY participants differing in depression-load, we used ARMA, a well-validated approach to understand and forecast timeseries, followed by *k*-means clustering. ARMA-defined COBY participants with the greatest past depression-load relative to the two other ARMA-defined COBY groups and HC showed significantly greater activity during emotional regulation task performance in a visual social cognition and salience perception^22–26^ network comprising right temporoparietal junction, right insula, left fusiform gyrus, and bilateral precuneus. Given that the activity in almost none of these regions was associated with present-scan-day symptom severity, this pattern of abnormally elevated activity likely reflects greater attention to socially salient emotional face distracters in the task in COBY participants with greater past depression-load, rather than participants with greater present-scan-day symptom severity. While all COBY participants performed the task accurately, such an aberrant pattern of neural activity might compromise emotional regulation in more complex everyday social contexts in BD individuals with more severe past depression-load. Indeed, we show that average interpersonal relationship assessment on the A-Life is positively related to right insula activity (see Supplementary information). Greater precuneus activity was reported in BD vs. healthy adults during social emotional task performance^23,27^; and deficits in social cognition, including perspective taking, theory or mind, and empathy, were previously reported in adults and youth with BD^28–30^, although the extent to which past depression-load accounted for these previous findings is unclear. Taken together, our present findings and these previous reports suggest that deficits in social cognition might be related to the impact of past depression-load on neural activity in social processing networks.

Distinct patterns of abnormally elevated neural activity in BD-COBY participants vs. HC during emotional regulation task performance were observed in left dlPFC, left hippocampus, and right somatosensory cortex; and activity in these regions was positively correlated with affective, but not with anxiety, symptom severity. These regions are implicated in emotional processing and regulation, with dlPFC implicated in more effortful appraisal and reappraisal strategies, hippocampus supporting implicit context-related reappraisal processes^31^, and right somatosensory cortex implicated in emotion processing and experience of emotions^32,33^. Our findings also parallel reports of greater left dlPFC^34–36^ and hippocampal^36^ activity during emotional regulation in adults and youth with BD-type-I vs. adults with BD-type-II and healthy adults, and vs. healthy youth, respectively; and abnormally elevated resting state functional connectivity in the somatosensory cortex in adults with BD-types-I and II^37^. Abnormally elevated activity in these regions might thus reflect either a compensatory recruitment of these regions to facilitate emotional regulation, or aberrant appraisal (and reappraisal) of face stimuli and heightened emotional experience during task performance, predisposing to the development of depressive and manic symptoms in BD. While it is not possible to distinguish between these two possible explanations, it is striking that activity in these regions was not associated with past history of depression, indicating that this pattern of abnormal dlPFC, hippocampal and somatosensory cortical activity is more likely to be related to present pathophysiological processes rather than effects of previous depression in BD. The left-sided focus of dlPFC and hippocampal activity might reflect the less efficient attentional processing capacity of left vs. right hemispheres^38^, resulting in more inefficient emotional regulation-related redirection of attention during the task.

Abnormal activity common to both main analyses was observed in regions involved in emotion processing and communication preparation, including right thalamus^39^, premotor cortex^39^, and bilateral cerebellum^40^. There were also positive relationships among present-scan-day affective symptoms and activity in right thalamus and bilateral cerebellum that distinguished ARMA-defined COBY groups and HC. Thus, abnormal activity in these regions might reflect aberrant processes involved in emotion processing and communication preparation that are associated with the cumulative burden of both long-term depression and present affective and anxiety symptom severity in BD.

COBY participants taking psychotropic medication showed lower, rather than higher, activity in regions showing main effects of group in the main analyses. Thus, it is unlikely that our findings were confounded by effects of medication. Similarly, there were no significant relationships between activity in the vast majority of regions showing main effects of group in each of the main analyses and age of illness onset, previous comorbid disorders, past mania, hypomania or anxiety severity, or past hypomania, mania and anxiety history loads. The absence of associations between amygdala activity and past depression-load or present-scan-day BD symptoms was an unexpected finding, but might reflect the emotional regulation nature of the paradigm, with between-group differences in activity reflecting aberrant recruitment of neural circuitries related to this process, rather than amygdala-centered circuitry supporting emotional processing per se. The COBY sample is relatively modest, and, with only one neuroimaging assessment, we were not able to examine neural changes accompanying symptom trajectories. Additionally, treatment across the sample was heterogeneous, and there were unbalanced numbers of participants with BDI and BDII. While future studies can aim to replicate our present findings, the COBY sample is, however, a unique cohort of well-characterized adults with pediatric-onset BD, who have been clinically followed for up to 17.5 years.

We show distinct patterns of aberrant neural activity related to long-term effects of depression vs. those related to present BD symptoms in a unique sample of adults with BD with well-characterized long-term past depression-load. Our findings can help guide targeting of future neuromodulation interventions to networks associated specifically with underlying pathophysiological processes of BD, rather than to those networks in which abnormal activity reflects long-term illness effects.

## Supplementary information

Supplement
