# Supplementary material for: Emotional regulation neural circuitry abnormalities in adult bipolar disorder: dissociating effects of long-term depression history from relationships with present symptoms
Source: Transl Psychiatry. 2020 Nov 2;10:374. doi: 10.1038/s41398-020-01048-1 (PMC7608654; doi:10.1038/s41398-020-01048-1)
Supplement: Supplementary file 1 — Supplement [file 41398_2020_1048_MOESM1_ESM.docx]

**Online-only Supplements**

**Methods**

*Data repository*

COBY data are uploaded to the NIMH Data Archive. The collection ID is 2392

*Exclusion Criteria*

Study exclusion criteria were: systemic medical illnesses, neurological disorders, history of trauma with loss of consciousness, use of central nervous system affecting non-psychotropic medications, IQ<70 assessed by the Wechsler Abbreviated Scale of Intelligence (WASI), positive drug/alcohol screen on the day of MR scan, alcohol/substance abuse in the past 3 months (determined by the K-SADS-PL), significant visual disturbance, non-English speaker, autistic spectrum disorders/developmental delays, pregnancy, claustrophobia, and ferromagnetic metal in the body. Participants were excluded from the analysis for excessive head movement, [1] data acquisition artefact, incomplete data acquisition, and past PSR data incomplete for >4 weeks before scan day (n=26), leaving 54 COBY participants (Age=18.9-32.7 on scan day) Table 1. Included and excluded participants did not differ on age, gender or IQ.

*Power*

The sample size of 80 for this study provided 75- 87% power to detect an effect size of f=.30 to .35 for two groups and 58- 73% power to detect an effect size of f=.30 to .35 for four groups using one way ANOVA.

*Description of A-LIFE assessment*

The A-LIFE assessment has two components 1. ongoing DSM‐IV psychiatric disorders and 2. onset of new DSM-IV disorders since the previous evaluation using the A‐LIFE Psychiatric Status Rating (PSR) scale. The COBY study assessed for the full complement of DSM-IV diagnoses. For the present study, we focused on depression and hypo/mania, as these are the primary symptoms of BD. At each follow‐up interview, the interviewer reviewed the participant's reported symptoms from the last interview, and then, using identifiable anchor points during the interval period such as memorable dates or events (e.g., holidays, school start and end dates, etc.), probed for symptoms that were present at that time. The interviewer then probed for subsequent changes in symptomatology. Although the PSR ratings are made on a week‐by‐week basis, the participant is not asked how they were feeling during each week; instead, the PSR scores change only if there is an identified ‘change point’ in the frequency, duration, or level of impairment associated with a symptom or disorder. Each score continues until an identified ‘change point’. For the depression and hypo/mania scores used in the present study, PSR scores ranged from 1-6; 1-2=no or minimal symptoms, to 3–4=varying levels of subthreshold symptoms and impairment, and 5 and 6=full DSM‐IV criteria, with 6 as the most severe and impaired. The assessment was administered to adolescents and parents separately; however, if the child had difficulty recalling symptom or mood changes, and were <14 years old, he/she was interviewed with a parent or guardian. Discrepancies between informants’ ratings were discussed by clinical staff. Summary scores were based on all available information.

All assessments were completed by trained research staff and were presented to child psychiatrists/psychologists for diagnosis and rating confirmation. If needed, participants’ medical records were obtained and reviewed for additional information. The A‐LIFE intraclass correlations for mood disorders in this study were ≥0.8 ^13^.

*Present medication use and past medication trajectories.*

Present medication use was recorded as taking/not taking each class of medication mood stabilizer, antipsychotic medication, antidepressant medication, and stimulants.

Long term exposure to medications was evaluated in using *PROC TRAJ* in SAS. This procedure derives group-membership based on a mixture of probability distributions^13^ for each medication class of psychotropic medications (Lithium, Non-lithium mood stabilizers, Antipsychotics, Antidepressants, Stimulants).

*Neuroimaging Data Acquisition*

Neuroimaging data for the main analysis were collected on a 3T Siemens Prisma MRI scanner at the University of Pittsburgh Medical Center (UPMC). Anatomical images covering the entire brain were acquired using an axial 3D

MPRAGE sequence (TE/TI/TR=3.17ms/800ms/1520ms; flip angle=8; 176 1mm-thick slices; matrix size=256x176;). fMRI data were acquired using a T2*weighted(TR/TE=1500/30ms), multiband (MB) gradient echo sequence with a resolution of 2.3mm3 (FOV=220x220, matrix=96x96, =54 slices with 122 mm coverage). To minimize potential slice contamination with MB acquisition, a moderate acceleration (3x) was used, with an adequate distance between adjacent, simultaneously excited slices and a FOV/3 shift. MB data was reconstructed using fully sampled pre-scan data and a slice-GRAPPA algorithm to separate the 3 aliased slices.

*Functional Imaging task*

The emotional n-back (EFNBACK) task is a modified version of the n-back working memory task (Ladouceur, Silk et al. 2009), and has been employed previously in studies of BD youth and adults as a measure of the ability to redirect attention away from emotional distracters during cognitive task performance, i.e, a measure of emotional regulation and present symptom-related emotional regulation neural activity in BD (Bertocci, Bebko et al. 2011, Kerestes, Ladouceur et al. 2011, Bertocci, Bebko et al. 2014).

The EFNBACK task consists of visually presenting a pseudorandom sequence of letters with participants responding to a pre-specified letter. The n-back task includes two memory load conditions: a no-memory load (0-back-e.g., press the button to “M”) and high memory load (2-back-e.g., press the button whenever the presented letter is identical to the letter present two trials back (L-X-L)) each with one of four emotional face distracter conditions (fearful, happy, neutral or no face distracter). The task comprises two, 7- min 4-sec runs, for a total of 24 blocks- presented in a pseudorandomized order. Each block includes 12 trials. Trial duration is 500ms. The inter-trial interval comprises a fixation cross (flanked with faces), and is jittered (mean duration=3500ms). Participants respond as quickly as possible with their index finger to the target letter. Brief instructions are presented on the screen for 4000ms at the beginning of each block. Detailed instructions are provided during task practice prior to the scanning session. Our analysis focused on the 2-back with emotional face distracters conditions. Incorrect trials were excluded from the analysis.

*Data Preprocessing*

Data were preprocessed using a combination of software packages (SPM, FSL, AFNI) implemented in Nipype.58 Data for each participant were realigned to the first volume in the time series to correct for head motion. Realigned BOLD images were then co-registered with the subject’s anatomical image. Distortion was of this image was corrected with a fieldmap, employing the FSL FUGUE package. The anatomical image was normalized to the MNI/ICBM 152 template using a non-linear transformation and segmented into separate tissue types. BOLD images were then transformed to the same space via the segmented structural image (the DARTEL method), at a resolution of 2 mm3 isotropic voxel size. BOLD images were corrected for activity spikes using the AFNI 3dDespike tool, normalized for intensity and then spatially smoothed with a FWHM of 6 mm, using FSL’s SUSAN adaptive smoothing method. Participants with motion >4mm were excluded.

*EFNBACK task behavioral data*

Accuracy of 70% was used. All participants accurately pressed the button (mean button press accuracy = .95 (76/80 button presses) and successfully completed the task with mean accuracy (SD)=.97(.04). COBY and Healthy participants did not differ in the number of button presses (t85)-=1.40, p= .167. Healthy participants were more accurate (0.98(.03) than were COBY participants (.95(.04), t(85)=2.26, p=.027).

*Autoregressive moving average*

Autoregressive moving average (ARMA) models provide a parsimonious description for understanding weakly stationary timeseries, meaning constant mean across time points and the covariance is dependent upon the lag of two timepoints. The AR parameter describes the memory of the timeseries with variables regressed on the lag of past variable in the timeseries. The MA parameter reflects the linear combination of error terms in the timeseries. We examined stationarity using the augmented dickey-fuller (adf) test in “tseries” package and visually inspected the inverse AR and MA roots plots. Not all of the timeseries were stationary. N=39 were stationary p<.05; n=5 were trend stationary (.06<p<.1), and n=10 were not stationary. We used differencing to correct the timeseries that were not stationary all timeseries that were not stationary were successfully corrected with differencing adf pvalue<.01. Given that it was our goal was not forecasting of individual timeseries but rather classification of the timeseries into groups, we standardized ARMA model (p,q) as (4,2) for each participant. The implication of this standardization increased the error for forecasting the individual participant model but does not adversely impact the relative AR parameters used for classification.

*Clinical differences*

The ARMA-defined groups differed on number of depressive episodes (F(2,51)=5.36, p=.008) with more episodes or Group-3 relative to Group-2 (p=.041) and Group-1 (p=.002), Group-2 did not differ from Group—1 (p=.501). The ARMA-defined groups differed on whether or not they reported a past depressive episode likelihood ratio chi2=9.34 df=2, p=.009, all 15 (100%) of the participants in Group-3 reported a previous depressive episode while 18/27 (66.7%) of the participants in Group-1 and 9/12 (75%) of participants in Group-2 reported having a depressive episode.

The three ARMA-defined-COBY groups did not differ on gender p=.985; Group-1: 13 male and 14 female; Group-2: 6 male and 6 female; Group-3: 7 male and 8 female; or age at scan F(2,51)=1.04, p=.359; mean age (standard deviation) Group-1: 25.3(4.0); Group-2: 27.1(4.2); Group-3: 25.1(3.8).

*K-means Clustering*

Two-groups: Group-1 n=41, Group-2 n=13; AR1 F(1,52)=55.22, p<.001; AR2 F(1,52)=39.06, p<.001; AR3 F(1,52)=.739, p=.394, AR4 F(1,52)=.104, p=.748.

Three-groups: Group-1 n=27, Group-2 n=12, Group-3 n=15; AR1 F(2,51)=41.20, p<.001; AR2 F(2,51)=38.54, p<.001; AR3 F(2,51)=15.45, p<.001, AR4 F(1,51)=3.64, p=.033

Four-groups: Group-1 n=11, Group-2 n=11, Group-3 n=14, Grou-p4 n=18 AR1 F(3,50)=33.44, p<.001; AR2 F(3,50)=46.83, p<.001; AR3 F(3,50)=17.0924.84, p<.001, AR4 F(3,50)=2.43, p=.076

Five-groups: Group-1 n=3, Group-2 n=12, Group-3 n=20, Group-4 n=3, Group-5 n=16; AR1 F(4, 49)=33.76, p<.001; AR2 F(4, 49)=50.09, p<.001; AR3 F(4, 49)=18.64, p<.001, AR4 F(4, 49)=3.67, p<.011.

**Exploratory Results**

**ARMA-defined-COBY group vs Healthy participants main effect of group results with present-scan-day medications and past medication trajectories**

Present-scan-day medication use:

There were no associations between present-scan-day antidepressant medication in conjunction with mood stabilizer medication, antipsychotic medication, and stimulant medication use and activity in any other clusters showing a main effect of group in this analysis (all ps>.123).

Historical medication trajectories:

ARMA-defined-COBY participants with a high/persistent historical use of lithium showed significantly lower activity in right thalamus (t(49)=2.39, p=.021. All other ps>.107.

ARMA-defined-COBY participants with a high/persistent historical use of antipsychotic medication showed significantly lower activity in right temporoparietal junction (t(49)=2.19, p=.033), right thalamus (t(49)=2.88, p=.006, right premotor cortex (t(49)=2.24, p=.029), left fusiform gyrus t(49)=2.55, p=.014) and left corpus collosum (t(49)=2.11, p=.040) and bilateral cerebellum (left: t(49)=2.30, p=.026; right: (t(49)=2.37, p=.022).

**Other past illness history variables**

In ARMA-defined COBY participants, there were no relationships between neural activity from the ARMA-defined-COBY group vs HC model and proportion of weeks with threshold manic (all ps>.125), or generalized anxiety symptoms (all ps>.291). There was a positive relationship between left fusiform activity and proportion of weeks with threshold hypomanic symptoms (r=.30, p<.028). There were no other significant relationships between neural activity in the 4-by-3 model and proportion of weeks with threshold hypomanic symptoms (all ps>.181). Supplemental table 3.

In ARMA-defined COBY participants, there were no relationships between neural activity from the ARMA-defined-COBY group vs HC model and age of onset of a mood disorder (all ps>.095).

In ARMA-defined COBY participants, activity from the ARMA-defined-COBY group vs HC model did not differ by lifetime comorbid history of ADHD (all ps>.125), generalized anxiety disorder (all ps> .105), conduct disorder (all ps>.062), or oppositional defiant disorder (all ps>.243).

In ARMA-defined COBY participants, past depression severity trajectories (all ps>.111) and mania severity trajectories (all ps>.125) were not related to activity in the ARMA-defined-COBY group vs HC model.

**Relationships with interpersonal relationship assessment near to scan**

The A-Life was administered as a monthly assessment (range 1-5) of interpersonal relationships. Average interpersonal relationships with parents, sibling, and boyfriend/girlfriend near to scan was positively related to activity in the visual and social cognition and salience perception network specifically, right insula activity (rho=.332, p=.024) in COBY participants. There were no other significant relationships: all ps>.105.

**BD-COBY vs Healthy participants main effect of group results with current medications and past medication trajectories**

Present-scan-day medication use:

Right premotor activity (t(51.6)=3.71, p=.001) was lower in COBY participants who were currently taking antipsychotic medication. There were no associations between present-scan-day antipsychotic medication use and activity in any other clusters showing a main effect of group in this analysis (all ps>.119). There were no relationships between present-scan-day antidepressant, mood stabilizer, or stimulant use and activity in any clusters showing a main effect of group in this analysis (all ps=.139).

Historical medication trajectories:

COBY participants with a high/persistent historical use of antipsychotic medication showed significantly lower activity in right thalamus (t(49)=2.72, p=.009), and left hippocampus (t(49)=2.35, p=.023), left cerebellum (t(49)=2.09, p=.041).

There were no relationships with history of lithium, non-lithium mood stabilizer, antidepressant, or stimulant medication use and activity in any clusters showing a main effect of group in this analysis (all ps>.077).

**Other past illness history variables**

Past illness load:

In BD-COBY participants, there were no relationships between neural activity from the BD-COBY vs. HC model and percentage of weeks with threshold manic (all ps>.181), or generalized anxiety symptoms (all ps>.305). There was a relationship between right cerebellum activity and proportion of weeks with threshold hypomanic symptoms (r=.29, p<.035). There were no other significant relationships between neural activity in the BD-COBY vs. HC model and proportion of weeks with threshold hypomanic symptoms (all ps>.129). Supplement table 4.

In BD-COBY participants, left dlPFC activity was negatively associated with age of mood disorder onset (r=-.30, p=.031). There were no other relationships between neural activity from the BD-COBY vs. HC model and age of mood disorder onset (all ps>.147).

Left cerebellum activity from the BD-COBY vs. HC model was higher in COBY participants with a history of comorbid ADHD (t(49)=-2.53, p<.015) with comorbid ADHD mean left cerebellum = -.47(.74) without comorbid ADHD = -.96(.46); all other region ps>.064. Left dlPFC activity was higher in COBY participants without a lifetime history of comorbid conduct disorder (mean=.03 (.43), with comorbid conduct disorder (-.56(.84)) (t(49)=2.46, p=.018; all other region ps>.071). Activity did not differ by lifetime comorbid history of generalized anxiety disorder (all ps> .099), or oppositional defiant disorder (all ps>.338).

In BD-COBY participants, there were no relationships between neural activity from the BD-COBY vs. HC model and past depression severity trajectories (all ps>094) and past mania severity trajectories (all ps>.130).

Relationships between past symptom trajectories and present symptoms

We calculated the correlations between present day scores and past trajectories and loads for each symptom dimension. Present day depression (HamD) was correlated with the past KDRS intercept (i.e., adjusted KDRS severity at the start of the study) (r=.318, p=.023) but not correlated with the past KDRS slope (r=.113, p=.430). Present day mania (YMRS) was correlated with the past KMRS intercept (r=.398, p=.004) and the past KMRS slope (r=.335, p=.016).

Present day symptom scores were not correlated with past symptom dimension loads (calculated as percentage of time with a threshold score of 5 or 6); present day mania (YMRS) was not correlated with past mania load (r=.189, p=.170); present day mania (YMRS) was not correlated with past hypomania load (r=-.058, p=.676); present day anxiety (STAI) was not correlated with past anxiety load (r=.258, p=.059).
